# Supplementary material for: Medical students in distress: a mixed methods approach to understanding the impact of debt on well-being
Source: BMC Med Educ. 2024 Aug 30;24:947. doi: 10.1186/s12909-024-05927-9 (PMC11363506; doi:10.1186/s12909-024-05927-9)
Supplement: Supplementary file 2 — Supplementary Material 2 [file 12909_2024_5927_MOESM2_ESM.pdf]

S1 Table. Summary statistics of people who identified as having high debt stress (-2), vs. low debt stress (-1 or 0).

	Level of Stress Associated with Medical School Debt	Overall	Low (-1 or 0)	High (-2)
N		2,771	2,176	595
MS Year	Pre-Clinical	1463 (52.8)	1146 (52.7)	317 (53.3)
	Clinical	561 (20.2)	437 (20.1)	124 (20.8)
	Gap Year/Other	134 (4.8)	102 (4.7)	32 (5.4)
	Post-Clinical	613 (22.1)	491 (22.6)	122 (20.5)
Gender	Male	919 (33.2)	734 (33.8)	185 (31.1)
	Non-Male	1850 (66.8)	1440 (66.2)	410 (68.9)
Marital Status	Never Married	2411 (87.1)	1902 (87.5)	509 (85.5)
	Divorced/Widowed	19 (0.7)	13 (0.6)	6 (1.0)
	Married	338 (12.2)	258 (11.9)	80 (13.4)
Disability	No	2481 (91.0)	1951 (91.3)	530 (90.0)
	Yes	245 (9.0)	186 (8.7)	59 (10.0)
URM	No	2391 (88.6)	1907 (89.6)	484 (84.6)
	Yes	309 (11.4)	221 (10.4)	88 (15.4)
Debt Burden	<$20K	728 (27.9)	691 (34.1)	37 (6.4)
	$20-100K	884 (33.9)	699 (34.5)	185 (31.8)
	>$100K	997 (38.2)	638 (31.5)	359 (61.8)
Specialty Competitiveness	Low	1309 (48.2)	1039 (48.6)	270 (46.6)
	Moderate	985 (36.2)	751 (35.1)	234 (40.4)
	High	424 (15.6)	349 (16.3)	75 (13.0)
Specialty Category	Surgical	413 (15.2)	326 (15.2)	87 (15.0)
	Medical	1414 (52.0)	1122 (52.5)	292 (50.4)
	Mixed (Surgical/Medical)	891 (32.8)	691 (32.3)	200 (34.5)
Degree Type	MD	2648 (95.8)	2094 (96.6)	554 (93.1)
	DO	115 (4.2)	74 (3.4)	41 (6.9)
School Category	Private	1409 (51.0)	1089 (50.2)	320 (53.8)
	Public	1354 (49.0)	1079 (49.8)	275 (46.2)
Region	Northeast	1019 (36.9)	817 (37.7)	202 (33.9)
	West Coast	718 (26.0)	547 (25.2)	171 (28.7)
	Non-Coastal	1026 (37.1)	804 (37.1)	222 (37.3)

City Character	Non-Metropolitan	1272 (46.1)	990 (45.7)	282 (47.6)
	Metropolitan	1485 (53.9)	1175 (54.3)	310 (52.4)
School Average Tuition	<$40K	365 (13.4)	299 (14.0)	66 (11.3)
	$40 - $60K	1745 (64.1)	1366 (63.8)	379 (65.0)
	>$60K	614 (22.5)	476 (22.2)	138 (23.7)
Leave of Absence for Wellbeing	Never Considered	2177 (78.8)	1747 (80.5)	430 (72.4)
	Considered	478 (17.3)	344 (15.9)	134 (22.6)
	Have Taken	109 (3.9)	79 (3.6)	30 (5.1)
